# Injectable Hydrogel Guides Neurons Growth with Specific Directionality

**DOI:** 10.3390/ijms24097952

**Published:** 2023-04-27

**Authors:** Yun-Hsiu Tseng, Tien-Li Ma, Dun-Heng Tan, An-Jey A. Su, Kia M. Washington, Chun-Chieh Wang, Yu-Ching Huang, Ming-Chung Wu, Wei-Fang Su

**Affiliations:** 1 Department of Materials Science and Engineering, National Taiwan University, Taipei 10617, Taiwan; 2 Department of Surgery, University of Colorado Anschutz Medical Campus, Aurora, CO 80045, USA; 3 National Synchrotron Radiation Research Center, Hsinchu 30076, Taiwan; 4 Department of Materials Engineering, Ming Chi University of Technology, New Taipei 24301, Taiwan; 5 Department of Chemical and Materials Engineering, Chang Gung University, Taoyuan 33302, Taiwan; 6 Center for Green Technology, Chang Gung University, Taoyuan 33302, Taiwan; 7 Division of Neonatology, Department of Pediatrics, Chang Gung Memorial Hospital at Linkou, Taoyuan 33305, Taiwan

**Keywords:** hydrogel, polypeptide, cellulose nanofiber, injectable, aligned structure, neuron, tissue engineering, three-dimensional tomography, calcium imaging

## Abstract

Visual disabilities affect more than 250 million people, with 43 million suffering from irreversible blindness. The eyes are an extension of the central nervous system which cannot regenerate. Neural tissue engineering is a potential method to cure the disease. Injectability is a desirable property for tissue engineering scaffolds which can eliminate some surgical procedures and reduce possible complications and health risks. We report the development of the anisotropic structured hydrogel scaffold created by a co-injection of cellulose nanofiber (CNF) solution and co-polypeptide solution. The positively charged poly (L-lysine)-r-poly(L-glutamic acid) with 20 mol% of glutamic acid (PLLGA) is crosslinked with negatively charged CNF while promoting cellular activity from the acid nerve stimulate. We found that CNF easily aligns under shear forces from injection and is able to form hydrogel with an ordered structure. Hydrogel is mechanically strong and able to support, guide, and stimulate neurite growth. The anisotropy of our hydrogel was quantitatively determined in situ by 2D optical microscopy and 3D X-ray tomography. The effects of PLLGA:CNF blend ratios on cell viability, neurite growth, and neuronal signaling are systematically investigated in this study. We determined the optimal blend composition for stimulating directional neurite growth yielded a 16% increase in length compared with control, reaching anisotropy of 30.30% at 10°/57.58% at 30°. Using measurements of calcium signaling in vitro, we found a 2.45-fold increase vs. control. Based on our results, we conclude this novel material and unique injection method has a high potential for application in neural tissue engineering.

## 1. Introduction

The digital revolution has changed eye-use habits, causing negative consequences for ocular health. Long-term use of electronic products has been reported to increase intraocular pressure (IOP) [1,2], forcing the elongation of the optic axis, which then compresses the cornea and optic nerve. An increase in IOP is often accompanied by a variety of orbital disorders such as keratoconus or glaucoma [3]. Studies have also pointed out that the annual myopia rate is increasing, with a particular effect upon the world’s younger generations [4,5]. Clinical studies confirm that high myopia can increase the incidence of glaucoma [6]; the morbidity rate of glaucoma has indeed increased annually in recent surveys [7]. Currently, glaucoma is the most common cause of blindness, second only to cataracts [8]. In primary closed angle glaucoma, the patient is born with an anatomically narrow drainage angle. This can restrict the outflow of the aqueous fluid and elevate IOP. Additionally, the displacement of the lens caused by phubbing and mydriasis resulting from a lack of light or tight ciliary muscles associated with eye tension can cause aberrant aqueous humor drainage. Ischemia, secondary to elevated IOP, causes retinal ganglion cell (RGC) death and degeneration of the optic nerve, which is comprised of RGC axons [9]. Therefore, blindness caused by optic nerve damage is a permanent and irreversible condition with no current treatments available to patients [10,11].

Neural tissue engineering (NTE) was first proposed in 1987 as a promising technology to regenerate damaged CNS neurons and their axons [12]. The concept of tissue engineering combines three main elements: cells, engineering tissue architecture, and engineering materials [13,14]. A potential benefit of using tissue engineering in medical applications is relief from organ or tissue recovery and transplantation. Instead, damaged or missing organs and tissues can be cultivated and used to repair, replace, or reconstruct defects. Engineered 3D tissue architecture that mimics the extracellular matrix can be designed with different functions to match the intrinsic characteristics of the target tissue; for example, the high-frequency contraction/relaxation of cardiomyocytes [15,16] and the specific directional communication of nerves [17,18]. To reestablish proper nervous system signaling, engineered neural must incorporate specific directionality to guide the orderly growth of nerves [19]. Thus, it is the objective of this research to develop injectable hydrogel under shear force that can promote the neurite growth with directionality and signal transport functionality in a biomimetic extracellular.

Previously electrospinning scaffolds, hydrogels, and hybrid scaffolds are commonly used in engineered tissue architecture. Although electrospinning scaffolds can effectively guide the growth of neurites, studies have found that cells grown on stiff surfaces may cause unusual adhesion [20,21]. Thus, we chose hydrogels as our tissue engineering architecture for their mechanical properties, which are similar to those of living organisms. Additionally, hydrogels have a bio-friendly high water-content structure, which can be made for injectable delivery, reducing the risks associated with surgical transplantation [22]. One potential issue is that the high water content of hydrogels causes difficulty in obtaining highly ordered and stable structures. Anisotropic hydrogels can be arranged through self-assembled peptides (SAPs), electromagnetic field induction, or stress–strain induction. The advantage of SAPs hydrogels arises from the utilization of natural amino acids or peptide sequences with beneficial properties such as the IKVAV sequence taken from Laminin, which can improve cell attachment when applied. Unfortunately, its low synthesis yield causes high material costs and difficulties in obtaining long-range arrangements make the SAPs difficult to apply clinically [23,24]. Electromagnetic field-induced hydrogels are limited by high costs despite the technique’s efficiency for obtaining high-ordered structures. Additionally, high electromagnetic fields are required to align the molecules that present hidden dangers to cells in 3D culture [25,26,27,28,29]. 

Instead, we elected to orient our hydrogels using simple and low-cost shear force via syringe injection to align the molecules [30,31,32,33,34,35,36]. The repeated monomeric units of glucose can self-arrange cellulose chains into the large and ordered structure of cellulose microfibril [37], which can be easily aligned under shear force. Thus, we selected the easy-to-align nanocellulose fiber (CNF) as one of the components in our hydrogels. We confined the molecules of hydrogels in a long (5.7 cm) and small diameter needle (22 Gauge: 0.168 mm I.D.) and capillary tube (9 cm long and 0.2 mm O.D.) to minimize the stress differences between the wall and center of the syringe device.

**Figure 1:**
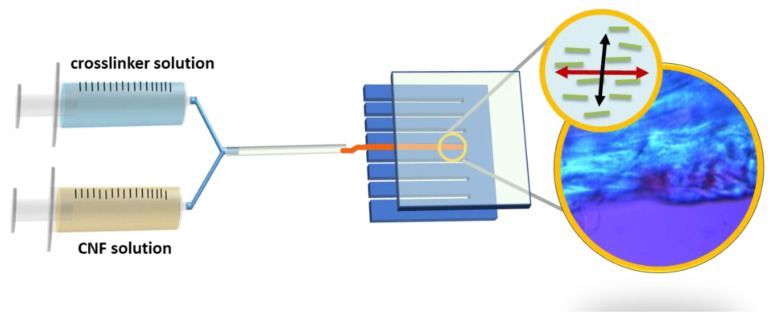
Schematic diagram of the preparation of the anisotropic-structured hydrogel via shear force using co-injection of CNF and crosslinker solutions.

CNF contains negative charges which quickly crosslink with cation poly-L-lysine-based crosslinkers through static charge attraction and hydrogen bonding. The poly-L-lysine-based crosslinkers were selected for their anti-inflammatory properties and cell/protein adhesion properties (which create a neuroprotective and neurotrophic environment), and are amenable to neural tissues and nerve-like cells used in research such as the transformed rat cell line, PC12 and human ARPE-19 cells [38,39,40,41]. Poly-L-lysine was further modified to copolymerize with the excitatory neurotransmitter, L-glutamic acid, to improve the growth and differentiation of the neurites [17,18,42,43,44]. Figure 1 shows the schematic diagram of the preparation of anisotropic structured hydrogel.

The parallel needle walls of a co-injection syringe were used to align the CNF and crosslinker solutions, respectively. Then, the two-shear aligned individual solutions were combined through a capillary tube to form a hydrogel. The diffusion gradient causes a second stage arrangement from within the hydrogels. The shear stress applied to the hydrogels by the wall of the capillary tube during injection provided a third stage alignment coinciding with the second stage arrangement. A polarized optical microscope (POM) and transmission X-ray microscope (TXM) were used to analyze the alignment structures of our hydrogels in the micro- and nano-scales, respectively. They are the techniques to examine the morphologies of hydrogels in situ without using high vacuum techniques such as SEM or TEM which would distort the wet gel structures [45]. PC12 cells are regularly used as a model of sympathetic neurons and neuronal differentiation [46]. We performed in vitro experiments to demonstrate that PC12 neurites were guided by the ordered hydrogel structures. Ca^2+^ is utilized in this study and others as an intracellular messenger that controls many cellular responses in various excitable and non-excitable cell types [47]. We used calcium imaging in ARPE-19 cells to demonstrate an enhanced responsiveness of ocular tissue derived cells cultivated on our scaffolds [48]. The combination of natural and synthetic materials holds tremendous potential for developing injectable hydrogels with enhanced biocompatibility, reduced cytotoxicity, and improved mechanical properties [49,50,51,52]. This study demonstrates the successful crosslinking of negatively charged CNFs with positively charged copolypeptides into hydrogels. The copolypeptides contain a moiety of lysine and glutamic acid that promote nerve axon growth and a water solubility that represents a breakthrough in hydrogel material for neural tissue engineering.

## 2. Results and Discussion

**Figure 2:**
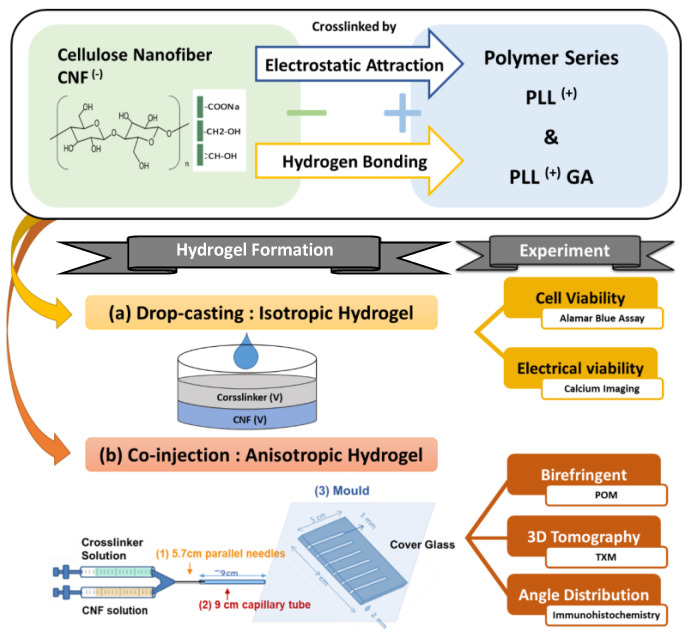
Schematic diagrams of hydrogel preparation (**a**) isotropic gel and (**b**) anisotropic gel.

**Figure 3:**
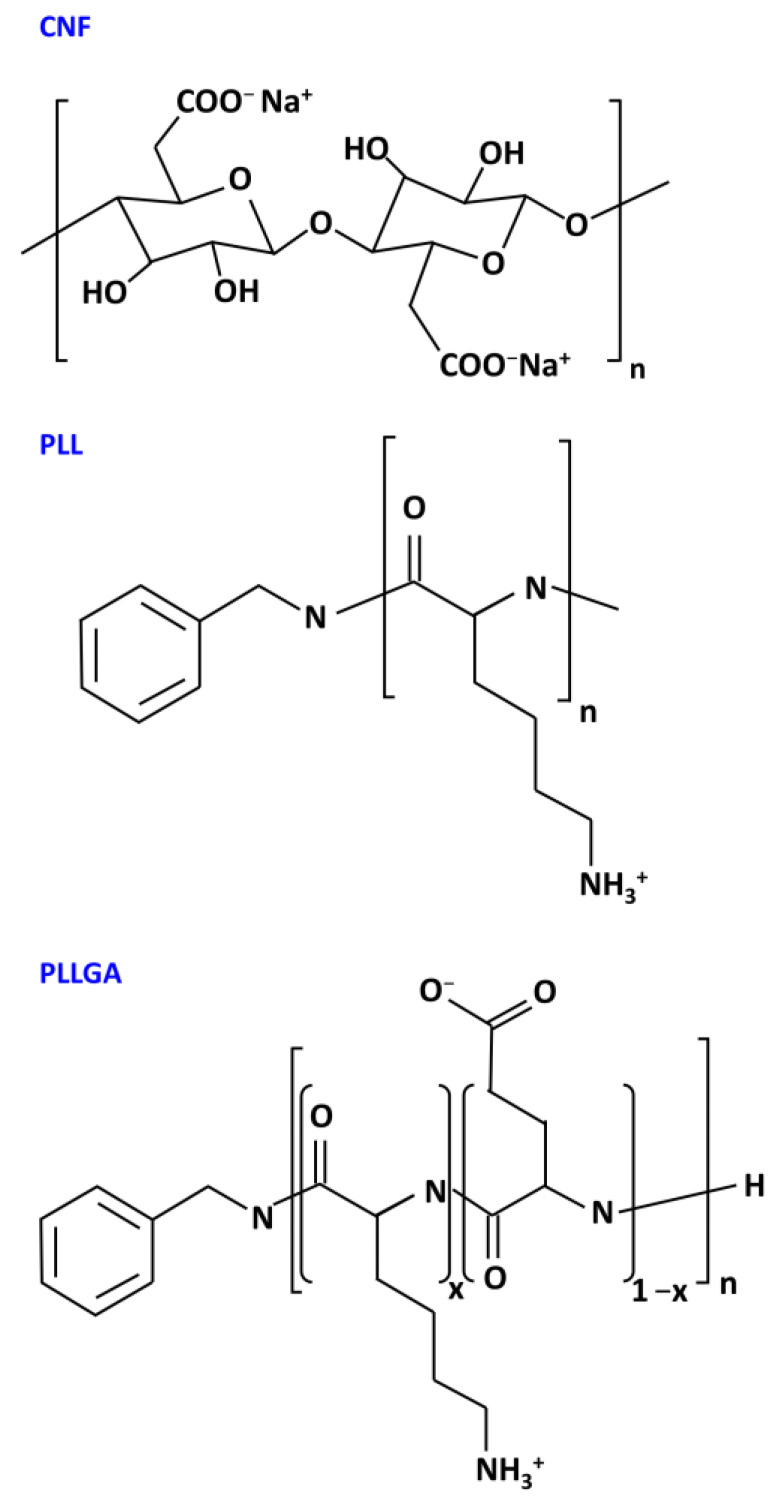
Chemical structures of CNF, PLL, and PLLGA in the hydrogel.

Hydrogels were prepared by blending aqueous solutions of CNF and polylysine-based crosslinkers PLL and PLLGA through injection methods as depicted in Figure 2 and described above in Materials and Methods. The chemical structures of CNF, poly-L-lysine-based, and the crosslinkers (PLL and PLLGA) are shown in Figure 3. The crosslinkers were synthesized in our laboratory accordingly to established methods [45].

**Figure 4:**
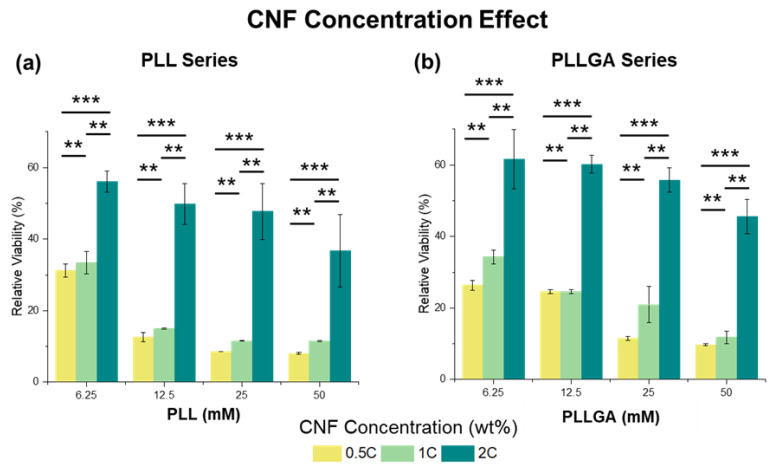
The CNF concentration effect in (**a**) PLL and (**b**) PLLGA crosslinker series on the relative viability from AlamarBlue assay on Day 6 PC12 cells. *n* = 7. The Kruskal–Wallis H-test was used to determine significant differences between groups. (** = *p* < 0.01, *** = *p* < 0.001).

To determine an appropriate hydrogel composition for neurite growth study, we first considered the biotoxicity of the scaffolds by quantifying cell viability using AlamarBlue assay, a redox indicator for cell proliferation. PC12 cells were seeded onto the isotropic hydrogels using two kinds of poly-L-lysine-based, crosslinkers (PLL and PLLGA). For PLL crosslinked CNF hydrogel, on Day 6, the relative cell viability plummeted to less than 20% at 0.5C and 1C samples for all PLL concentrations tested (6.25 mM to 12.5 mM), as shown in Figure 4a. This result demonstrates that PLL exhibits biotoxicity at high dose.

**Figure 5:**
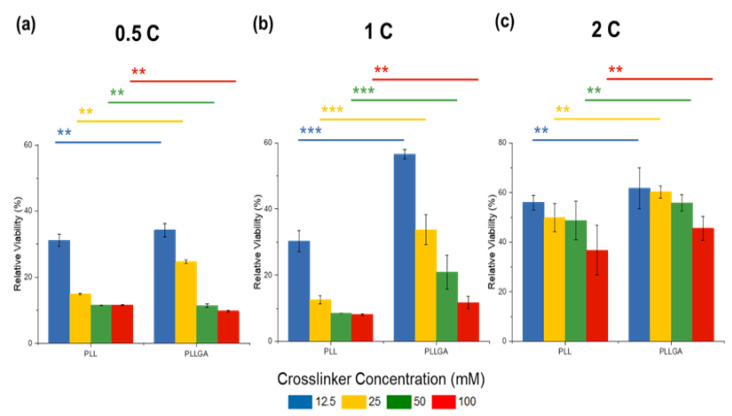
The enhancement of the cell viability on Day 6 through the modification from PLL to PLLGA by varying the CNF concentration (**a**) 0.5 wt.%, (**b**) 1.0 wt.%, and (**c**) 2.0 wt.%. *n* = 7. The Kruskal–Wallis H-test was used to determine significant differences between groups. (** = *p* < 0.01, *** = *p* < 0.001).

However, the negative impact of PLL crosslinker can be offset and cell viability improves when CNF solution concentration is increased to 2C. Previous studies report that glutamate, an endogenous peptide that acts as an excitatory neurotransmitter, can promote not only cell viability, but also neural function [17,53]. To address potential cytotoxicity, PLL was modified by copolymerizing 20 mol% L-glutamic acid to L-lysine to obtain PLLGA (Figure 4b). Of note, this addition of L-glutamic acid reduces the positive charge of L-lysine accordingly. Thus, the actual crosslinker molar concentration of PLLGA was higher than the equivalent molar concentration of the crosslink sites. Despite the higher actual crosslinker molar concentration of PLLGA, we observed increased cell viability at equivalent crosslink sites (Figure 5). Significant differences in viability reduction were seen between PLL and PLLGA under the same effective concentration (*p* < 0.001). Therefore, the modification of PLL by glutamic acid (PLLGA) was an effective method to increase cell viability when using poly-L-lysine-based crosslinkers. Next, in vitro experiments were performed using the optimized 2C-6.25PLL and 2C-6.25PLLGA to represent the PLL series and PLLGA series, respectively.

**Figure 6:**
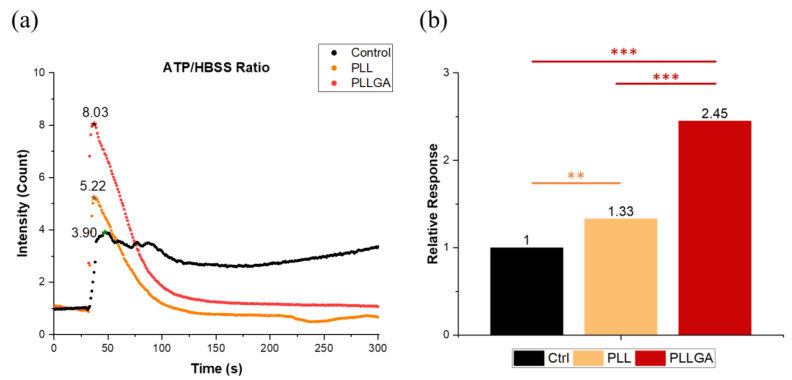
(**a**) The fluorescent intensity profile of the calcium indicator to ATP stimulation and (**b**) the electroactivity of ARPE cultured on 2C-6.25PLL and 2C-PLLGA on Day 1 was represented as the response peak heights which were normalized with the control group. *n* = 7. The Kruskal–Wallis H-test was used to determine significant differences between groups. (** = *p* < 0.01, *** = *p* < 0.001). The star sign marks the highest point of each curve.

Neuronal communication, like other secretory cells communication, depends on ligand engagement of receptors on the cell membrane, often coupled to ionic channels whose activation and ability to change voltage potential is conditionally dependent on the linkage between site and gating by ATP occupancy [54,55]. ARPE-19 cells have been used extensively to characterize Ca^2+^ signaling and transport mechanisms [56,57,58]. Phototransduction by the neural retina rod and cone photoreceptors utilizes Ca^2+^ dynamics by modulating the cGMP-gated channels and cGMP turnover [59]. Moreover, the retinal pigmented epithelium (RPE), which is vital for the nourishment of the retina, depends on Ca^2+^ as a second messenger to maintain homeostasis [60]. To investigate the effects of our hydrogels on neural signaling in the setting of the ocular system, we imaged calcium signaling in adult retinal pigment epithelial cell line-19 (ARPE-19), which have apically oriented cilia that are widely used in researching retinal pathology, particularly macular degeneration [61,62,63,64]. RPE cells are known to release ATP, and stimulating adenosine receptors regulate RPE cells interaction with photoreceptors [64]. We found that the intracellular Ca^2+^ concentration increased in ARPE19 cells after the extracellular dosing of ATP. This could result from the direct influx of Ca^2+^ via ATP-dependent calcium channels or a release from intracellular stores, possibly mediated by P2Y_1_ receptor activation [65]. The fluorescence intensity peak of the calcium indicator can correlate with electroactive neuronal behavior. As shown in Figure 6, hydrogels with polypeptide-based crosslinkers (PLL and PLLGA) had a significant improvement compared with the control cells cultured on the coverslip. We attribute this to the well-established neurotrophic effects of lysine [38,39,40]. PLLGA with 20 mol% glutamic acid had the larger effect on Ca^2+^ response compared with the PLL series. Glutamic acid is an excitatory neurotransmitter that can additionally activate ionotropic glutamate NMDA receptors [66]. This could account for the drastic enhancement of activation signals associated with PLLGA over PLL. Furthermore, these results suggest ARPE-19 cells are highly electroactive on the glutamate containing hydrogel PLLGA.

After using Ca^2+^ imaging and AlamarBlue viability assays to determine the optimal scaffolds for cell culture on isotropic hydrogels, we examined whether aligned cellulose nanofibers could guide the orderly growth of NGF stimulated PC12 neurites. A scanning electron microscope (SEM) is typically used to confirm engineered nano-scale structures; however, in this case, the high vacuum environment of SEM complicates the analysis of aqueous samples. Hydrogels with over 90% water content need to be dried, which might cause an unexpected deformation or collapse due to the loss of swelling by water. Instead of SEM, POM and TXM were used to characterize the aqueous samples under 2D micro-scale and 3D nanoscale, respectively. Due to the high aspect ratio of CNF, the optical properties of the long axis and the short axis of CNF are significantly different, which makes it an optically anisotropic material. POM, which can analyze the birefringence, is appropriate for characterizing the orientation of fiber in hydrogels. We found the resolution to be insufficient for characterizing the hydrogel at the nanoscale of neurite structure. Freeze-dried samples can be analyzed by SEM, but it will lose their original nanofibrous hydrogel structure. Wet hydrogel samples can be analyzed by TEM, but its procedure is tedious and high cost. Therefore, TXM which can resolve down to 60 nm using high coherence synchrotron radiation was employed to confirm hydrogel directed neurite anisotropy. Samples with very small contrast can be resolved by leveraging the phase shift associated with RuO_4_ staining. Even so, it is still difficult to distinguish between water and hydrogels composed of light elements of carbon, hydrogen, oxygen, and nitrogen in the hydrogels under TXM. To address these challenges, it was necessary to increase the concentration of crosslinkers to enhance signal contrast. So, for the structure characterizations by POM and TXM, the high concentration crosslinker samples, 2C-50PLL and 2C-50PLLGA were used instead of those determined to be optimal for the in vitro work (2C-6.25PLL and 2C-6.25PLLGA). 

**Figure 7:**
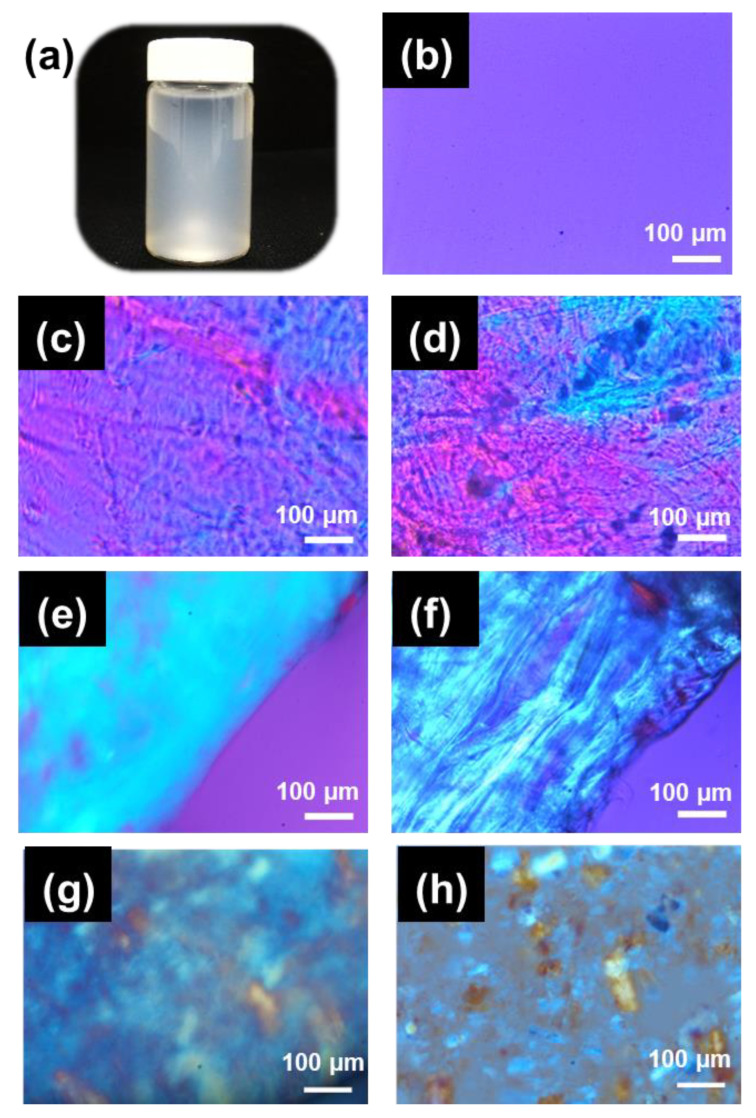
(**a**) Typical photo image of CNF hydrogel. POM images of the well-dispersed (**b**) 2.0 wt.% CNF solution, (**c**) iso-2C-50PLL, (**d**) iso-2C-50 PLLGA, (**e**) ani-2C-50PLL, (**f**) ani-2C-50 PLLGA, and anisotropic hydrogels, (**g**) iso-2C-50PLL, and (**h**) iso-2C-50 PLLGA, which were fabricated with not well-dispersed CNF solution showed the micro-scale aggregation bundle.

A typical CNF hydrogel photo image was shown in Figure 7a. A uniform magenta POM image of the well-dispersed 2.0 wt.% CNF solution was captured under cross-polar mode with the compensator filter inserted as depicted in Figure 7b. Figure 7c,d POM shows that crosslinking the peptide-based crosslinkers (PLL or PLLGA) produces some random anisotropic regions, likely due to shrinkage during gelation. The random anisotropic areas were unable to guide neurite extension with consistent orientation (alignment). Comparatively, the anisotropic hydrogels with persistent directionality successfully guided neurites along their axes over increased distances. The birefringence of ani-2C-50PLL and ani-2C-50PLLGA with the shear stress was applied to align the fibers emerged as a uniform blue along with the 1st and 3rd quadrant. The blue region represents the constructive retardation effect due to two coincident ordinary axes of compensator and specimen. Thus, we verified the creation, functionality, and uniform structural orientation using the co-injection method for CNF hydrogels at a submicron scale, as shown in Figure 7e,f. Despite these data, additional research should be carried out in the future to demonstrate that the hydrogel fibers are orientated along the long axis and capable of supporting regenerative neurite guidance over long distances, such as for peripheral nerves and for optic nerve from the retina, across the optic chiasm, and then to properly target the visual centers within the brain’s cortex.

Since CNF has a large amount of hydrogen bonds, it easily aggregates to form a micro- or even larger-scale cellulose bundle. The CNF has to be well dispersed without any aggregation in the solution using an ultrasonic oscillator before crosslinking with polypeptide, as shown in Figure 7b. The ordinary axis of the CNF can be derived from POM images of the hydrogels formed by aggregation CNF solution, as shown in Figure 7g,h. The compensator is a positive crystal. When inserted into the optical path along a northwest-southeast direction, the ordinary axis was along a northwest–southeast direction. CNF was derived to be a positive crystal as the aggregate bundles along with northwest–southeast present blue. The aggregation images also demonstrated that the arrangement applied by the shear stress became less effective when the CNF was poorly dispersed.

**Figure 8:**
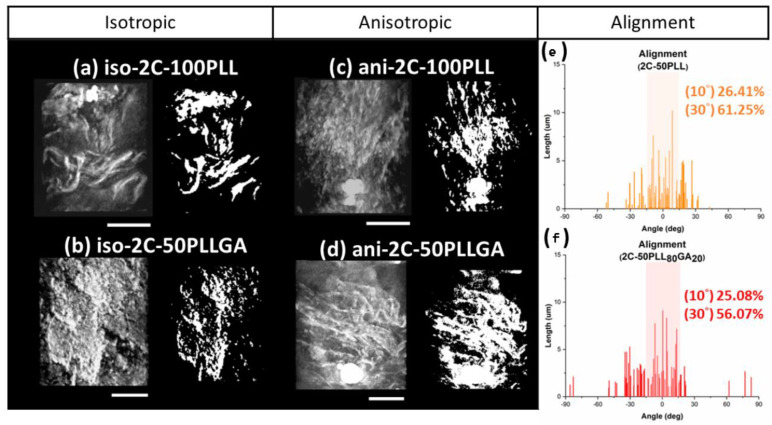
The TXM image of (**a**) iso-2C-50PLL, (**b**) iso-2C-50PLLGA, (**c**) ani-2C-50PLL, and (**d**) ani-2C-50PLLGA, and the alignment of hydrogel molecules was performed by angle distribution analysis from (**e**) ani-2C-50PLL and (**f**) ani-2C-50PLLGA. (*n* = 50), scale bar 5 micron.

We also considered that fibers in the hydrogel were arranged in a 3D space, while the 2D POM images only show the azimuth angle distribution projected on the focus plane. In this case, the fiber distribution at different angles of elevation was ignored. As a result, the reported orientation could be overestimated. That is why using TXM with the capacity for submicron resolution was essential for characterizing the 3D orientation. This in situ analysis for the hydrogels was completed under an ambient environment that prevented any deformation due to dehydration. The structure of the isotropic hydrogels presented sheet-like porous crosslinked features, with multiple groups of small anisotropic regions appearing randomly in the 3D models obtained by TXM images acquired at different angles, as seen in the left column of Figure 8a,b. This result is consistent with the observation in the POM study. The anisotropic hydrogels prepared by the co-injection method presented here were arranged in the long axis direction, as shown in Figure 8c,d. Additionally, the middle of the hydrogels presented larger-scale fiber bundles, which can reach tens to hundreds of nanometers, and lengthen out to 15 μm across the entire observation area (15 μm × 15 μm). The mechanism behind the formation of this structure is speculated to be the driving force of diffusion at the solution interface that occurs when the two solutions in the capillary tube encounter one another. We hypothesize the diffusion gradient forces molecular chains to present an arrangement of parallel interfaces.

The extent of fiber alignment in the hydrogel was analyzed using TXM images acquired under different rotation angles. The left column of Figure 8a–d shows the 3D images of different hydrogel samples. The alignment structure of the hydrogel gel under shear force can be seen in the left column of Figure 8c,d. The 3D images were transformed into 2D black and white 2D images through Fourier transformation, as shown in the right column of Figure 8a–d. Short segments (down to tens of nanometers) and long segments across the entire observation area coexisted in the TXM images; thus, the angle distribution of fibers was calculated by weighting the length to the angle. The weighted average of the angles was redefined as the axis of orientation. The statistically significant results are shown in Figure 8e,f. The alignment of Ani-2C-50PLL and Ani-2C-50PLLGA was 26.41% at 10°*/*61.25% at 30° and 25.08% at 10°*/*56.07% at 30°, respectively, as the degree of alignment was defined as the proportion of angles between −5°~5° and −15°~15°. The average of the alignment is calculated by weighted average due to nanofiber bundle. The single nanofiber under the TXM image may be formed by several CNF nanofibers, so weighted average is more accurate than simple number average.

The extent of alignment was calculated by statistical analysis. We found that our co-injection method aligned fibers on the surface, making contact with the syringe and capillary tube walls. Additionally, we found consistent long-range arrangement in a 3D space. The 3D tomography model exhibits a complex network of pores which will be useful for future 3D cell culture work. Such pores can allow oxygen, nutrients, and waste material diffusion and exchange in biological settings. Importantly, the fiber structural organization is not disjointed, but rather, parallel. It is possible that neuronal dendritic arborization might be enhanced by the complex interlaced fiber features. The highly plexiform high-order branching of neural dendrites arises from complex, nonlinear interactions between neural signals [67]. As the size and complexity of dendrites decrease, the activity in neurons is attenuated [68]. The loss of nerve complexity may be a precursor to neurodegeneration [69]. Thus, an engineered increase in the dendritic complexity might play a crucial role in neural protection. 

**Figure 9:**
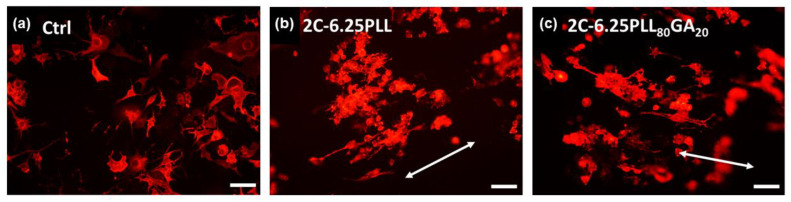
Representative images of immunohistochemical staining results of PC12 cells cultured on (**a**) untreated glass coverslip and (**b**) 2C-6.25PLL, (**c**) 2C-6.25PLL80GA20 anisotropic hydrogel for five days. The arrow bar indicates the directionality of the neurites.

**Figure 10:**
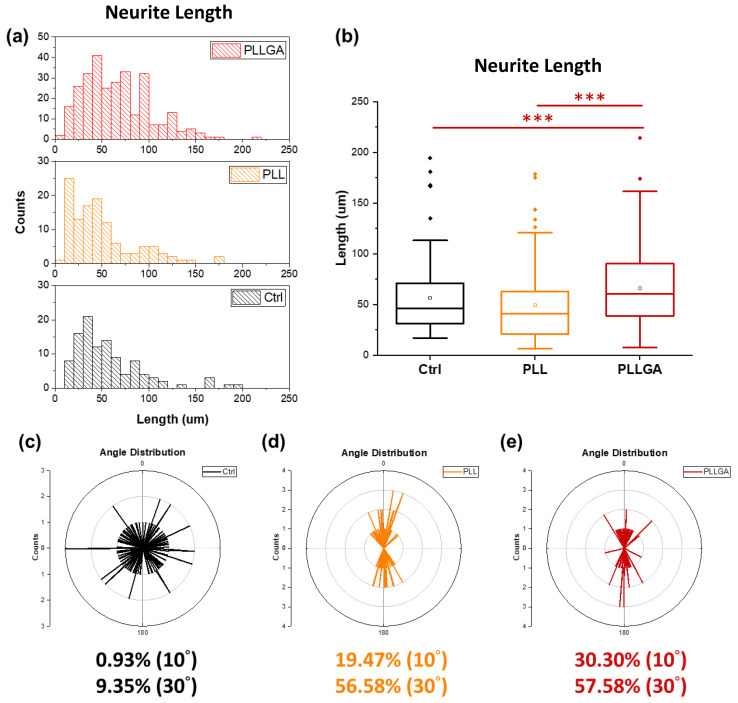
(**a**) The neurite length distribution and (**b**) the box chart of the neurite length. The % alignment of (**c**) control group, (**d**) ani-2C-6.25PLL, (**e**) ani 2C-6.25PLLGA. The counts (*x*-axis) represent the “number of the neurites”. Additionally, 10 degrees represent neurite growth between +10 degree and −10 degree. The Kruskal–Wallis H-test was used to determine significant differences between groups. (*** = *p* < 0.001).

On Day 5 after seeding, the cytoskeleton of PC12 cells stimulated with 2.5s-NGF was stained to investigate whether anisotropic hydrogels could successfully guide neurites growth with specific directionality as shown in Figure 9. Nerve growth and differentiation were determined by measuring neurite length distribution, as shown in Figure 10a. Neurite length distribution presented a positive Skew distribution, which was consistent with the distribution of normal nerve growth [38]. The average neurites length on the coverslip was 59.62 μm, while the ani-2C-6.25PLL and ani-2C-6.25PLLGA can reach 59.38 μm and 69.37 μm, respectively, as shown in Figure 10b. A 16% increase in neurite length is achieved using ani-2C-6.25PLLGA hydrogel scaffold. Typically, cells have difficulty adhering and growing on soft material substrates [70]. Here, neurites differentiation upon ani-2C-6.25PLL is comparable with growth on the coverslip control group. One explanation for this is that the high mechanical properties of the peptide-crosslinker-based hydrogels enhance cellular adhesion [45]. Another rationale is that the lysine in PLL mimics the endogenous neuroprotective role in the CNS [71,72,73]. Regardless, the addition of the 20 mol% glutamic acid significantly increased neurite length, presumably due to stimulation by glutamate, a neurotransmitter used by various neurobiological systems including the neural retina [74]. The results of our neuronal cell differentiation study corroborate the results of this study’s calcium signal imaging (Figure 6). The low doses of polypeptide crosslinkers effectively promoted the electroactivity and differentiation performance in cultured neuronal cell lines. Among them, the 2C-6.25 PLLGA with 20 mol% glutamic acid copolymerized with lysine exhibits significant and outstanding performance.

The extent of alignment of neurites guided by the anisotropic hydrogels was determined using polar plot (detailed in Section 3.5.3). Cultured cells on the untreated glass coverslips were typically radial in morphology (Figure 9a). The alignment was only 0.93% in the range of ±5°, and 9.35% in the range of ±15° (Figure 10c). In contrast, neurites grown on an ani-2C-6.25 PLL and an ani-2C-6.25 PLLGA were 19.74% at 10°*/*56.58% at 30° and 30.30% at 10°*/*57.58% at 30°, respectively, as shown in Figure 10d,e. Thus, the anisotropic hydrogels fabricated by the co-injection method show great potential for use in effectively guiding nerve growth in a bipolar direction in both crosslinker systems.

## 3. Materials and Methods

### 3.1. Materials

The polylysine-based crosslinkers, PLL and PLLGA, were synthesized by ring opening reaction of their corresponding N-carboxyanhydrides [45]. CNF was synthesized as previously described [75]. Chemicals for the characterization of the hydrogel structure are ruthenium tetroxide, 0.5% stabilized aqueous solution RuO_4_(aq), (20427-56-9, Polysciences Inc., Warrington, DC, USA), and Au nanoparticles (AC11-500-CIT-DIH-100-1-EP, Nanopartz, Loveland, CO, USA). Reagents for in vitro experiments are as follows: AlamarBlue™ cell viability reagent (DAL1025, Thermofisher, Waltham, MA, USA), nerve growth factor-2.5S from the murine submaxillary gland (NGF, N6009-4X25UG, Sigma, St. Louis, MI, USA), rhodamine phalloidin reagent (AB125138, Abcam, Cambridge, MA, USA), 4′,6-diamidino-2-phenylindole (DAPI, D1847, Sigma-Aldrich, St. Louis, MI, USA), Hank’s balanced salt solution, (1×), with calcium, magnesium, without phenol red (HBSS, SH30588, HyClone, Logan, UT, USA), Fluo-2 AM, green fluorescent Ca^2+^ binding dye (ab142775, Abcam, Cambridge, MA, USA), adenosine triphosphate (ATP, A2383, Sigma-Aldrich, St. Louis, MI, USA), Dulbecco’s modified eagle medium (DMEM/F12 1:1, SH30023.02, HyClone, Logan, UT, USA), and RPMI 1640 with L-glutamine (SH30011.02, HyClone, Logan, UT, USA).

### 3.2. Nomenclature

Cellulose nanofiber is abbreviated as (CNF). The CNF solution is abbreviated as C; the prefix before “C” represents the weight concentration of the CNF solution. Abbreviations for the two kinds of polypeptide crosslinker, poly-L-lysine, poly-(L-lysine)-γ-poly-(L-glutamic acid) are PLL and PLLGA, respectively. The prefix before each crosslinker indicates the effective molar concentration of the crosslinker. The nomenclature of the hydrogels is denoted by the composition of CNF solution and the crosslinker. The prefixes iso- and ani- represent the isotropic and anisotropic hydrogels, respectively. For instance, ani-1C-25PLL is an anisotropic hydrogel fabricated with 1.0 wt% CNF and 25 mM PLL.

### 3.3. Hydrogel Preparation

The CNF has to be well dispersed without any aggregation in solution using ultrasonic oscillator before crosslinking with polypeptide. Isotropic hydrogels were fabricated by drop-casting a mixture of equal volume of crosslinker solution (concentration ranged from 6.25–50 mM PLL, PLLGA or Ca^2+^) and CNF solution (~1.5 wt%), as shown in Figure 2a. After 24 h of aging, isotropic hydrogels were then prepared. Similar solution concentrations were used for anisotropic hydrogel preparation. The anisotropic hydrogel was placed in the capillary tube for 30 min in order to prevent chain relaxation before crosslinked. While anisotropic hydrogels were prepared by co-injecting the crosslinker solution and CNF solution through the applicator tip, dual cannula (SA-3605, FibriJet, Micromedics, Ventura, CA, USA), and gelled in the capillary tube (34507, Kimble, Vineland, NJ, USA), which was connected at the front end of the co-injector, as shown in Figure 2b. Aligned hydrogels were placed in a mold, then soaked in DI water to avoid water evaporation, preventing changes in the swelling ratio of hydrogels.

### 3.4. Characterization of Hydrogel Morphology

#### 3.4.1. Polarized Optical Microscopy

A polarized optical microscope (POM, DM 2500 M, Leica, Wetzlar, Germany) was used to study the micro-scale birefringence of all hydrogels. Hydrogels were placed on a glass slide (22-310397, Fisher Scientific, Waltham, MA, USA). The samples were observed under cross polar mode at 100× with the compensator filter inserted into the optical path. Hydrogels were kept moist by applying 1–2 drops of DI water during the measurement to prevent any structural changes due to water evaporation.

#### 3.4.2. Transmission X-ray Microscope

Hydrogels were stained with RuO_4_ (aq.) for 24 h, then any residual dye was removed with 1X DI water wash, and then Kapton tape (P-222 AMB, Nitto, Osaka, Japan) that was cut into triangles was used to affix samples onto the sample holder. Long thin samples around 100 μm were preferentially selected using the needle to avoid signals that were perpendicular to the rotation axis. Such signals could block the target imaging area. Next, a 5 μL Au nanoparticle suspension with a particle size around 400–500 nm was dropped on the sample as a calibration object. Samples were then sent into the transmission X-ray microscope (TXM, NSRRC 01B1, Taiwan) for imaging. After rotating samples from −75° to 75°, a 3D model of the hydrogel nanostructure was created by Fourier transformation of the 151 TXM images. 

### 3.5. In Vitro Studies Using Hydrogel

#### 3.5.1. Cell Viability 

AlamarBlue Assay was employed to study PC12 cell viability at 2, 4, and 6 days in vitro (Day 2, 4, 6) when 2D cocultured on top of the different compositions of hydrogels. The hydrogel gel sample was prepared by drop-casting a mixture of 180 μL crosslinker solution and 180 μL CNF solution in 48-well TC cell culture plate. The PC12 cells (Passage 9) were a gift from Prof. Jia-Shing Yu, Department of Chemical Engineering, National Taiwan University. The coverslip was not coated by any ECM molecules, nor was the hydrogel. PC12 cells were cultured in RPMI-1640 with L-glutamine with 10 *v*/*v*% HS (16050122, Gibco, Rockville, MD, USA), 5 *v*/*v*% FBS (SH30071, HyClone, Logan, UT, USA), 1 *v*/*v*% PSA (A5955, Sigma-Aldrich), and 1.0 g sodium bicarbonate (144-55-8, Sigma-Aldrich). Cells were incubated under 5% CO_2_ at 37 °C and sub-cultured with trypsin EDTA (0.5%, 03-051-5C, Biological Industries, Haemek, Israel). PC12 cells were seeded into sterile polystyrene (PS) 48-well TC cell culture plate (353078, Corning Falcon, New York, USA) which had been UV sterilized overnight. Cells grown on 12 mm coverslip (0111520, Marienfeld, Germany) were used as the control group, while coverslips sterilized with UV (24 h) without cells served as the blank group. After staining for 4h with 10 *v*/*v*% AlamarBlue kit diluted with 37 °C DMEM (21063-029, Gibco, Rockville, MD, USA), stained cell suspensions were transferred to a sterile PS 96-well treated cell culture microplate (351172, Corning Falcon, New York, NY, USA) for detection by a microplate detector (800TS, BioTEK, Winooski, VT, USA) to analyze the absorbance at 570 nm and 600 nm wavelength, respectively. The absorbance was converted into a reduced percent by Equation (1) to represent the cell viability [76].

(1) $$\mathrm{Percent}\;\mathrm{reduced}\;\left(\%\right)=\frac{\varepsilon_{\mathrm{ox}}\lambda_{595}A_{\lambda 570}-\varepsilon_{\mathrm{ox}}\lambda_{570}A_{\lambda 595}}{\varepsilon_{\mathrm{red}}\lambda_{570}{A^{\prime }}_{\lambda 595}-\varepsilon_{\mathrm{red}}\lambda_{596}{A^{\prime }}_{\lambda 570}}$$

λ_1_ = 570 nm; (ε_ox_) _λ1_ = 80,586; (ε_RED_) _λ1_ = 155,677

λ_2_ = 600 nm; (ε_ox_) _λ2_ = 117,216; (ε_RED_) _λ2_ = 14,652

A: Absorption of tested sample
A^′^: Absorption of blank

#### 3.5.2. Calcium Imaging

ATP stimulated calcium signaling was imaged 24 h after seeding cells (Day 1) on the 2D hydrogels. ARPE-19 cells (passage 37) were supplied by Dr. Ta-Ching Chen, National Taiwan University Hospital, Taipei, Taiwan. The cells were grown in DMEM/F12 1:1 medium with 10 *v*/*v*% FBS and 1 *v*/*v*% PSA (SV30010, HyClone, Logan, UT, USA). The cells were incubated under 5% CO_2_ at 37 °C and subcultured using trypsin EDTA for seeding at a density of 5 × 10^5^ cells/well into sterile PS 6-Well TC cell culture plate (353046, Corning Falcon, New York, NY, USA) which had been UV sterilized overnight. The hydrogel gel sample was prepared by drop-casting a mixture of 500 μL crosslinker solution and 500 μL CNF solution in a 6-well TC cell culture plate. Cells grown on the 22 mm coverslip (0111520, Marienfeld, Lauda-Konigshofen, Germany) were used as the control group. Calcium imaging was performed as previously described [77,78]. Fluo-2 AM was diluted with 37 °C HBSS to 5 μM and used to stain Day1 ARPE-19 for 30 min. We diluted 20 mM ATP with 37 °C HBSS to a final concentration of 1 mM to stimulate the cellular calcium signal. After removing the dye residues by 1.0 mL HBSS washing per well, an inverted fluorescent microscope (Eclipse Ti, Nikon, Tokyo, Japan) recorded the region of interest selected by Nikon NIS elements per second for 5 min. Next, 100 μL HBSS and 20 mM ATP solution were, respectively, added to the culture plates 30 s after staining to reveal changed induced by adding 1 mM ATP.

#### 3.5.3. Measurement of Length, Distribution, and the Extent of the Alignment of Neurites

NGF (100 ng/mL) was used to induce PC12 neurite growth. PC12 cells were seeded at a density of 1.6 × 10^4^ cells/well on the hydrogels in a 48-well cell culture plate. On Day 5, culture media was removed and then cells washed with 37 °C phosphate buffered saline (PBS, 1 tablet/160 mL H_2_O, SLCC8330, Sigma-Aldrich, St.Louis, USA) solution. Cells were fixed with 400 µL/well of 3.7 *v*/*v*% formaldehyde (37 wt% in water, 50-00-0, Acros Organics, Branchburg, NJ, USA) diluted with PBS. After 15 min of incubating at 37 °C under 5% CO_2_, fixative was removed and cells washed twice with PBS. A total of 400 µL per well of 1% Triton X-100 (X198-07, J.T. Baker, Phillipsburg, NJ, USA) diluted with PBS was used to permeabilize cells at room temperature for 10 min, then removed and washed using PBS. A total of 400 µL per well of 1 *w*/*v*% bovine serum albumin (BSA, 9048468, Sigma, St. Louis, MI, USA) was then added to PBS to pre-incubate for 60 min at room temperature to enhance staining. After removing the BSA/PBS pre-incubated solution, 300 units of rhodamine phalloidin dissolved in 1.5 mL methanol (67-56-1, Macron fine chemicals, Center Valley, PA, USA) was diluted 60 times in BSA/PBS. A total of 400 µL rhodamine phalloidin working solution was used to visualize the filamentous actin after 60 min of staining. The dye was removed and washed twice by BSA/PBS. Then, 400 µL of 300 nM DAPI solution diluted with BSA/PBS was used to label PC12 nuclei. After 15 min of staining, the dye was removed and cells washed twice by BSA/PBS. The samples were soaked in BSA/PBS to be imaged and measured. Neurite length and orientation were measured from the cell body to the end of the neurites by ImageJ. The average direction of the neurites was redefined as 0° for analysis of the neurite growth alignment on substrates. Three repeat measurements were performed for each experimental group, and over 50 neurites were measured in each sample.

### 3.6. Statistical Analysis 

The Kruskal–Wallis H-test was used to determine significant differences between groups. The *p*-values < 0.05, < 0.01, and < 0.001 are represented as significant, highly significant, and extremely significant, respectively, and are marked as *, **, and ***, accordingly.

## 4. Conclusions

We have successfully developed anisotropic-structured hydrogels by crosslinking negatively charged CNF with positively charged copolypeptides under shear force using a co-injection method. The arrangement of anisotropic hydrogels was characterized in situ by POM and TXM under micro- and nano-scale, respectively. We show the co-injected CNF hydrogels can enhance the extent of the alignment of NGF-stimulated neurite growth from 9.35% to 57.58% in the range of ±15°. We show a 2.45-fold increase over control in electroactivity of functional ATP-dependent Ca^2+^ signaling for ARPE-19 cells grown on our hydrogel. The cell viability of hydrogel is dependent on the amount of CNF and polypeptides, as well as the type of polypeptide. The cytotoxicity of hydrogel can not only be reduced, but its neurite growth performance is also increased by the incorporation of 20 molar % of glutamic acid moiety in polylysine as a co-polypeptide crosslinker: PLLGA. The cytotoxicity decreases with increasing amount of CNF and reducing amount of copolypeptide. The best biocompatibility of more than 60% cell viability is observed for the hydrogel sample of 2C-6.25PLLGA (2 wt% CNF and 6.25 mM PLLGA). We demonstrate a 16% increase in neurite length in stimulating directional neurite growth compared with the control using the optimal composition of hydrogel: 2C-6.25PLLGA. With this hydrogel, under shear force via co-injection method, an anisotropy of 30.30% at 10°/57.58% at 30° is reached. The injectable anisotropic-structured hydrogel reported here is a promising neural tissue engineering strategy for treating various neuropathies including those specifically related to the visual system and vision loss. The clinical use of injectable hydrogel has the potential advantage of avoiding open surgical procedure complications. 

## Data Availability

The data that support the findings of this study are available on request from the corresponding author.
